# A Bioengineered Nisin Derivative To Control Streptococcus uberis Biofilms

**DOI:** 10.1128/AEM.00391-21

**Published:** 2021-07-27

**Authors:** Mariana Pérez-Ibarreche, Des Field, R. Paul Ross, Colin Hill

**Affiliations:** a APC Microbiome Ireland, University College Cork, Cork, Ireland; b School of Microbiology, University College Cork, Cork, Ireland; University of Buenos Aires

**Keywords:** *Streptococcus uberis*, biofilms, NSR, antimicrobial agents, nisin A, nisin PV

## Abstract

Antimicrobial peptides are evolving as novel therapeutic options against the increasing problem of multidrug-resistant microorganisms, and nisin is one such avenue. However, some bacteria possess a specific nisin resistance system (NSR), which cleaves the peptide reducing its bactericidal efficacy. NSR-based resistance was identified in strains of Streptococcus uberis, a ubiquitous pathogen that causes mastitis in dairy cattle. Previous studies have demonstrated that a nisin A derivative termed nisin PV, featuring S29P and I30V, exhibits enhanced resistance to proteolytic cleavage by NSR. Our objective was to investigate the ability of this nisin derivative to eradicate and inhibit biofilms of S. uberis DPC 5344 and *S. uberis* ATCC 700407 (*nsr^+^*) using crystal violet (biomass), 2,3-bis-(2-methoxy-4-nitro-5-sulfophenyl)-2H-tetrazolium-5-carboxanilide (XTT) (viability) assays, and confocal microscopy (viability and architecture). When preestablished biofilms were assessed, both peptides reduced biofilm biomass by over 60% compared to that of the untreated controls. However, a 42% higher reduction in viability was observed following treatment with nisin PV compared to that of nisin A. Accordingly, confocal microscopy analysis revealed significantly more dead cells on the biofilm upper surface and a reduced thickness following treatment with nisin PV. When biofilm inhibition was assessed, nisin PV inhibited biofilm formation and decreased viability up to 56% and 85% more than nisin A, respectively. Confocal microscopy analysis revealed a lack of biofilm for *S. uberis* ATCC 700407 and only dead cells for *S. uberis* DPC 5344. These results suggest that nisin PV is a promising alternative to effectively reduce the biofilm formation of *S. uberis* strains carrying NSR.

**IMPORTANCE** One of the four most prevalent species of bovine mastitis-causing pathogens is *S. uberis.* Its ability to form biofilms confers on the bacteria greater resistance to antibiotics, requiring higher doses to be more effective. In a bid to limit antibiotic resistance development, the need for alternative antimicrobials is paramount. Bacteriocins such as nisin represent one such alternative that could alleviate the impact of mastitis caused by *S. uberis.* However, many strains of *S. uberis* have been shown to possess nisin resistance determinants, such as the nisin resistance protein (NSR). In this study, we demonstrate the ability of nisin and a nisin derivative termed PV that is insensitive to NSR to prevent and remove biofilms of NSR-producing *S. uberis* strains. These findings will add new information to the antimicrobial bacteriocins and control of *S. uberis* research fields specifically in relation to biofilms and *nsr*^+^ mastitis-associated strains.

## INTRODUCTION

Streptococcus uberis is an environmental Gram-positive bacterium belonging to the *Streptococcaceae* family and is one of the principal organisms responsible for bovine mastitis (1). Mastitis is considered one of the most frequent and costly diseases in the dairy industry, often resulting in production losses, culling, changes in product quality, and increased risk of other diseases, and its treatment requires time and money (2, 3).

One of the factors that contributes to the pathogenesis of Streptococcus uberis is its ability to form biofilm, communities of bacteria bound together by an extracellular polymeric matrix. Biofilms are common in nature, and it has been estimated that 99% of bacterial cells coexist in biofilm and only 1% live in a free or planktonic state (4). Bacteria in biofilms are in sessile form, exhibiting a different phenotype from the same cells in planktonic form, particularly concerning their growth rate and gene transcription (4). The extracellular matrix biofilm is composed principally of exopolysaccharides and water and a lower proportion of other macromolecules, such as proteins, DNA, and cell lysis debris (5). This matrix provides the bacteria with protection, making it difficult to remove, increasing its resistance to antibiotics, and making it impervious to host defenses (6, 7). Currently, antibiotics are the most widely used treatment to remove biofilms of *S. uberis* (8). However, a dramatic increase in antibiotic resistance poses a major threat to the treatment of infectious diseases, jeopardizing both antibiotic use and effectiveness. Consequently, this kind of therapeutic solution is not favorable at a time when an overall reduction in antibiotic use is advocated. This has led to the search for novel antibiotics that can be used as pharmaceuticals against pathogenic bacteria, such as *S. uberis*.

Among the potential alternatives to antibiotics are the bacterially produced lantibiotics, a family of polycyclic, ribosomally synthesized, and posttranslationally modified peptides that can inhibit the growth of many different bacteria. Within the family of lantibiotics, nisin is the most prominent member and has the ability to kill susceptible Gram-positive bacteria by binding the cell wall precursor lipid II and forming pores in the membranes (9). Nisin consists of 34 amino acids, with dehydrated residues (dehydroalanine and dehydrobutyrine), and five lanthionine rings that are crucial for stability and exhibits activity in the nanomolar range (10).

Recently, it has been shown that a gene cluster within *Streptococcus* strains encodes a nisin resistance protein (NSR) and an ABC transporter, NsrFP, both conferring resistance to nisin (11, 12). NSR has been shown to degrade nisin by cleaving the peptide bond between MeLan28 in-ring E and the serine at position 29. The resulting nisin 1 to 28 fragment has a significantly lower bactericidal efficacy and has reduced affinity for cell membranes (13). Several lantibiotic bioengineering strategies have been described that provide examples of how peptide functionality can be adapted significantly by the alteration of just one residue (14–17). Nisin derivatives can be generated using genetic engineering techniques by changing the amino acids in key positions of the NSR target to avoid its action.

Previous studies have demonstrated that a nisin derivative termed nisin PV with proline and valine substitutions at serine 29 and isoleucine 30, respectively, exhibits enhanced resistance to proteolytic cleavage by NSR (18). Therefore, in this study, we investigated the effects of nisin A (wild type [WT]) and nisin PV on the inhibition (prevention of biofilm formation) and eradication of biofilm (removal of established biofilm) of *S. uberis* NSR producers.

## RESULTS

### Nisin PV exhibits enhanced bioactivity against NSR-producing strains *S. uberis* DPC 5344 and ATCC 700407 by deferred antagonism assay.

**FIG 1:**
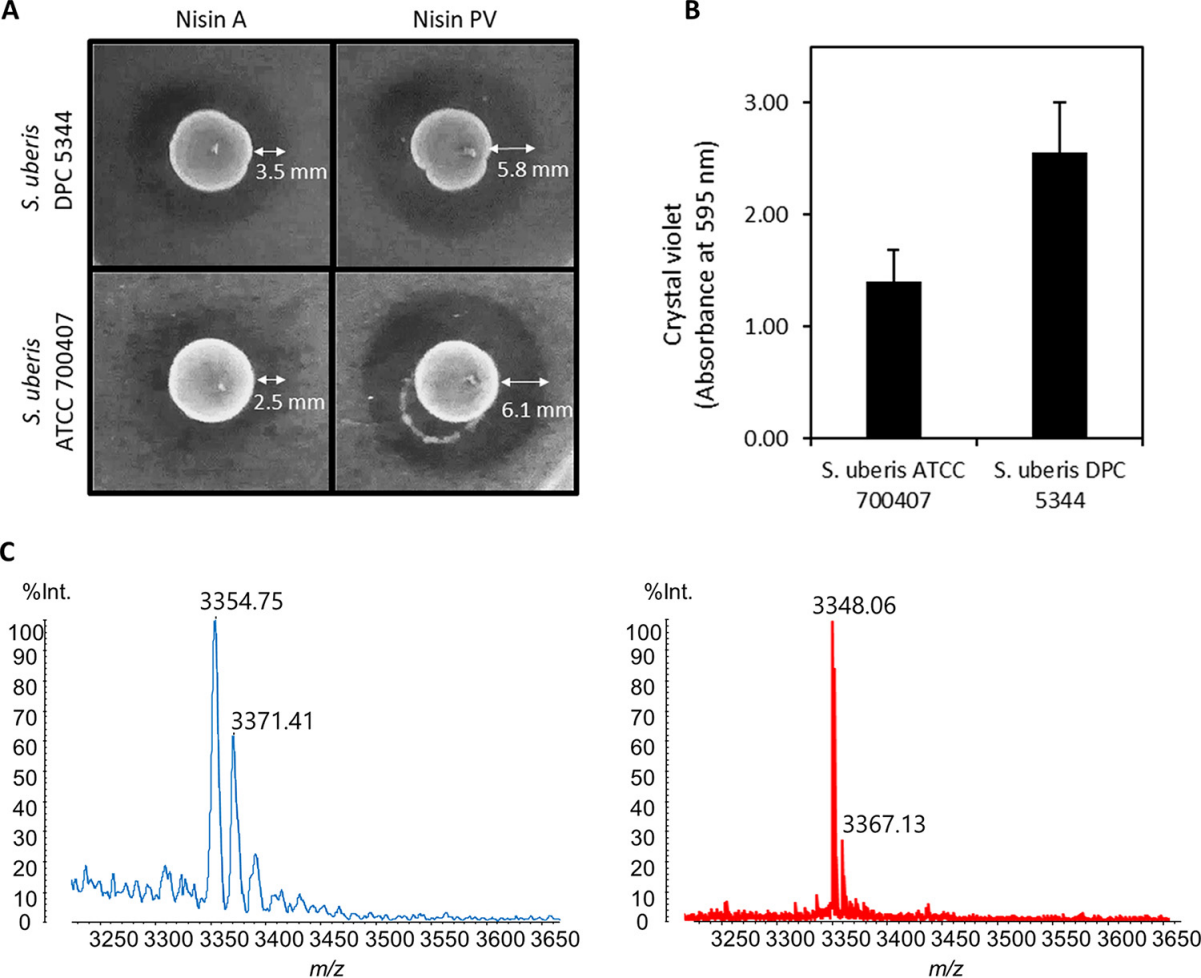
(A) Deferred antagonism assay of the L. lactis NZ9800(pCI372-nisA) nisin A-producing strain (wild-type control) and the nisin derivative PV producer strain L. lactis NZ9800(pCI372-nisA-PV) against the NSR^+^ strains *S. uberis* DPC 5344 and ATCC 700407. (B) Assessment of biofilm formation by *S. uberis* ATCC 700407 and DPC 5344. (C) Mass spectrometry analysis of nisin A (3,354.75 amu) and nisin PV (3,348.06 amu) after the purification process.

Bioactivity is defined as the inhibition zone in the overlay around the bacteriocin-producing strain as determined by the deferred antagonism assay. Nisin derivative PV displayed up to 60% enhanced inhibition of growth compared to that of nisin A against both NSR-producing strains, *S. uberis* DPC 5344 and ATCC 700407 (Fig. 1A).

### Biofilm formation ability of *S. uberis* DPC 5344 and ATCC 700407.

Given the clearly improved potency of nisin PV against planktonic cells of these strains, we proceeded to investigate its bioactivity on sessile cells following biofilm formation. The ability of these NSR-producing strains (18) to form biofilms was evaluated by crystal violet assays, the most commonly used technique to evaluate the capacity of a microorganism to develop biofilms. Both *S. uberis* DPC 5344 and *S. uberis* ATCC 700407 demonstrated a strong ability to form biofilms over the surface of the microtiter plate after 24 h of incubation at 37°C according to Stepanović criteria (19) (optical density cut-off value at 595 nm [ODc_595_] = 0.10). Particularly, *S. uberis* DPC 5344 displayed the hallmarks of a strong biofilm former as observed by the high absorbance value (optical density at 595 nm [OD_595_] = 2.56) (Fig. 1B). In contrast, *S. uberis* ATCC 700407 displayed lower biofilm formation (OD_595_ = 1.41) compared to *S. uberis* DPC 5344.

### Biofilm eradication activities of nisin A and PV against *S. uberis* strains.

**FIG 2:**
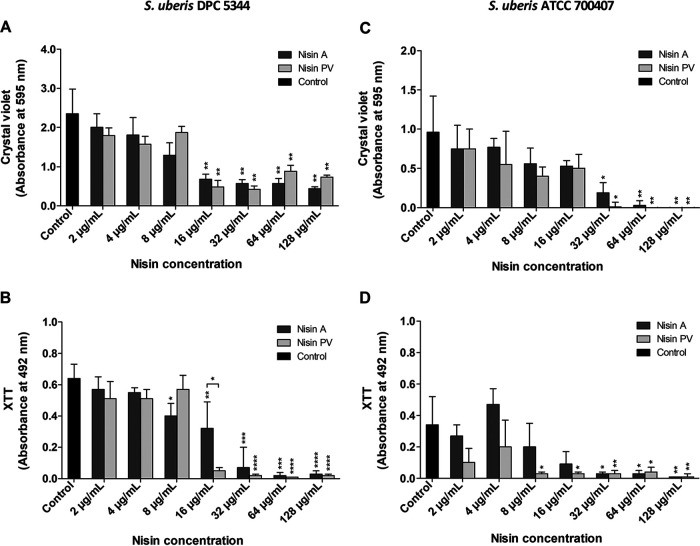
Evaluation of nisin peptides to eradicate *S. uberis* biofilms. *S. uberis* DPC 5344 (A, B) and *S. uberis* ATCC 700407 (C, D) biofilm treated with 2, 4, 8, 16, 32, 64, and 128 μg/ml of nisin A and PV for 24 h and evaluated for quantity and viability by crystal violet (A, C) and XTT (B, D) staining, respectively. The means and standard deviations of triplicate determinations are presented. Asterisks above each bar indicate statistically significant differences between pairwise comparison with the control group (Student’s *t* test). Asterisk above bracket indicates statistically significant differences between pairwise comparison between peptides used at the same concentration (Student’s *t* test) (*, *P* < 0.05; **, *P* < 0.01; ***, *P* < 0.001; ****, *P* < 0.0001).

Mass spectrometric analysis of the peptides was carried out to ensure purity (Fig. 1C). As expected, the maximum peak obtained for nisin A was at 3,354 atomic mass units (amu) and 3,348 amu for nisin PV. The concentrations of peptides employed to evaluate the efficacy of nisin A and PV against *S. uberis* biofilm cells were based on the MIC obtained for nisin A against planktonic cultures as previously determined (18). The MIC of nisin A on planktonic cells was equivalent for each strain, i.e., 16 μg/ml for *S. uberis* ATCC 700407 and *S. uberis* DPC 5344, but in the case of nisin PV, an 8-fold and 4-fold lower MIC was observed, respectively (2 μg/ml for *S. uberis* ATCC 700407 and 4 μg/ml *S. uberis* DPC 5344). Therefore, preformed biofilms in 96-well microtiter plates were evaluated following 24 h treatment with 2, 4, 8, 16, 32, 64, and 128 μg/ml of nisin A or PV, corresponding to 1/8×, 1/4×, 1/2×, 1×, 2×, 4×, and 8×, respectively, of the MIC value determined for the nisin A peptide against planktonic cultures. In the case of *S. uberis* DPC 5344, both nisin A and nisin PV had the capacity to eradicate established biofilms of this strain at a concentration of 16 μg/ml compared to the untreated control (*P* < 0.05) (Fig. 2A). Increasing concentrations did not appear to have any greater effect on biofilm removal as determined by crystal violet staining. However, when the viability of the cells was examined (Fig. 2B), a statistically significant reduction in biofilm viability (*P* < 0.001) was observed at 16 μg/ml of nisin PV compared to that of biofilms treated with the corresponding concentration of nisin A (*P* < 0.05).

In the case of *S. uberis* ATCC 700407, treatments with 32 μg/ml and higher with both nisin A and nisin PV had a statistically significant reduction in biofilm viability compared to that of the untreated control (Fig. 2C). Notably, however, nisin PV had a greater reduction on cell viability compared to that of nisin A for this strain from 8 μg/ml concentration (Fig. 2D).

### Biofilm formation inhibition activities of nisin A and PV against *S. uberis* strains.

**FIG 3:**
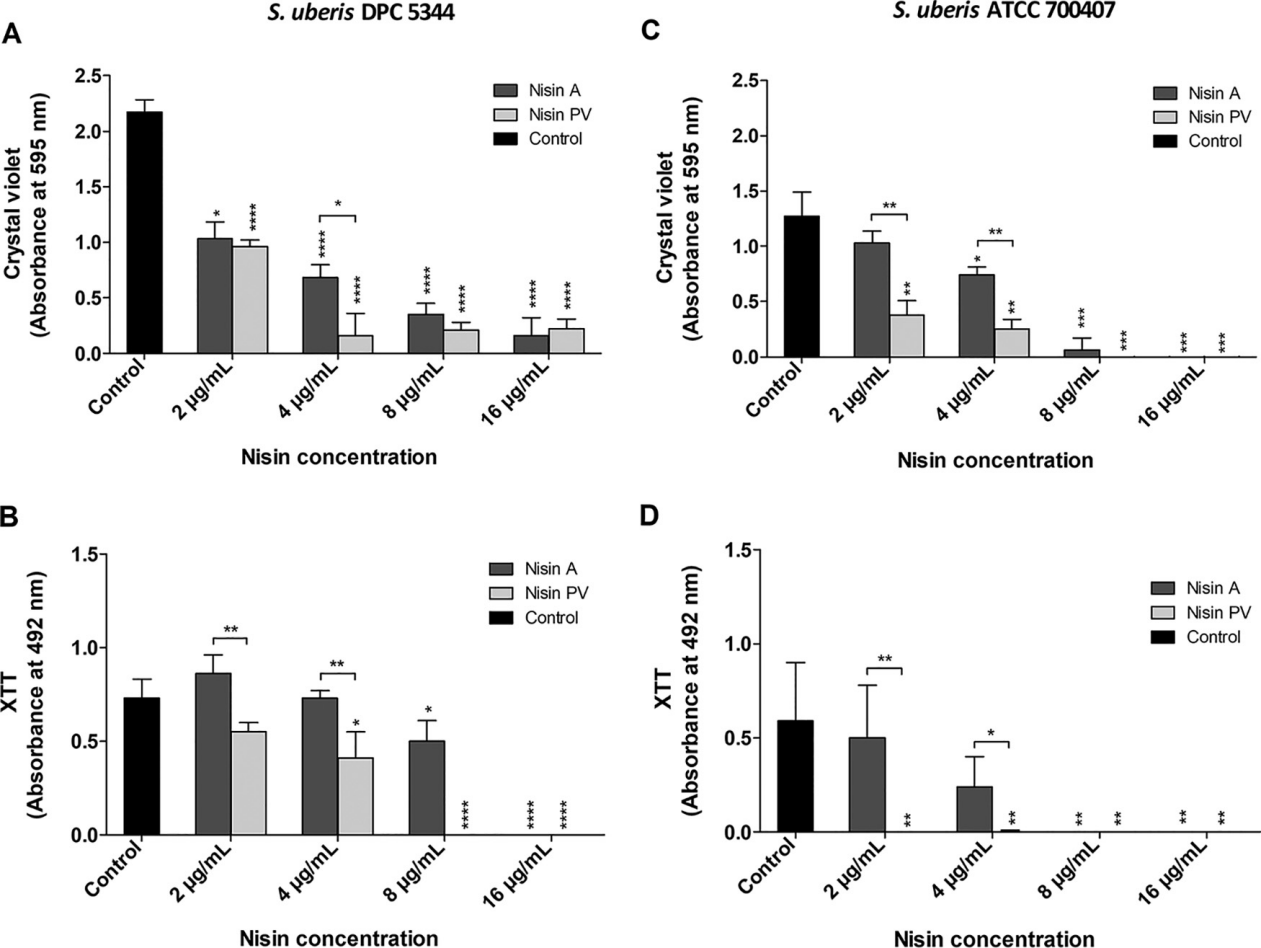
Evaluation of nisin A and PV to inhibit biofilm formation by *S. uberis*. *S. uberis* DPC 5344 (A, B) and *S. uberis* ATCC 700407 (C, D) biofilm treated with 2, 4, 8, and 16 μg/ml of nisin A and PV for 24 h and evaluated for quantity and viability by crystal violet (A, C) and XTT (B, D) staining, respectively. The means and standard deviations of triplicate determinations are presented. Asterisks above each bar indicate statistically significant differences between pairwise comparisons with the control group (Student’s *t* test). Asterisks above brackets indicate statistically significant differences between pairwise comparison between peptides used at the same concentration (Student’s *t* test) (*, *P < *0.05; **, *P < *0.01; ***, *P < *0.001; ****, *P < *0.0001).

For biofilm prevention studies, a 16 μg/ml and several dilutions thereof of nisin A and PV were tested against *S. uberis* strains. Following staining and absorbance readings at 595 nm, a 2-fold reduction in *S. uberis* DPC 5344 biofilm mass was observed in wells containing 2 μg/ml of both nisin peptides compared to the untreated control, but a significant difference (*P* < 0.05) in biomass reduction was observed in the presence of 4 μg/ml nisin PV compared to the same concentration of nisin A (Fig. 3A). When the viability of the *S. uberis* DPC 5344 biofilm was analyzed, even at the lowest concentrations tested (2 μg/ml, 4 μg/ml, and 8 μg/ml of nisin PV), a significant reduction in metabolic activity of 25%, 44%, and 100%, respectively, was observed compared to that of the same treatments with nisin A, which was only able to reduce the metabolic activity by 32% at 8 μg/ml (Fig. 3B). No metabolic cell activity was found at 16 μg/ml for both peptides (Fig. 3B). Furthermore, treatment with just 8 μg/ml nisin PV was sufficient to prevent the detection of viable biofilm cells compared to those treated with 8 μg/ml nisin A (Fig. 3B).

Regarding *S. uberis* ATCC 700407, nisin A and PV were capable of inhibiting biofilm formation at 8 μg/ml and 16 μg/ml. Remarkably, treatments with nisin PV revealed significant reductions in biofilm formation and biomass cell viability at concentrations of 2 μg/ml and 4 μg/ml compared to the treatment with nisin A (Fig. 3C and D). In addition, nisin PV was able to completely inhibit the presence of viable cells on the biofilm at all concentrations tested (Fig. 3D).

### Architecture and viability of *S. uberis* biofilm after nisin treatment for the removal of established biofilm evaluated by CLSM.

**FIG 4:**
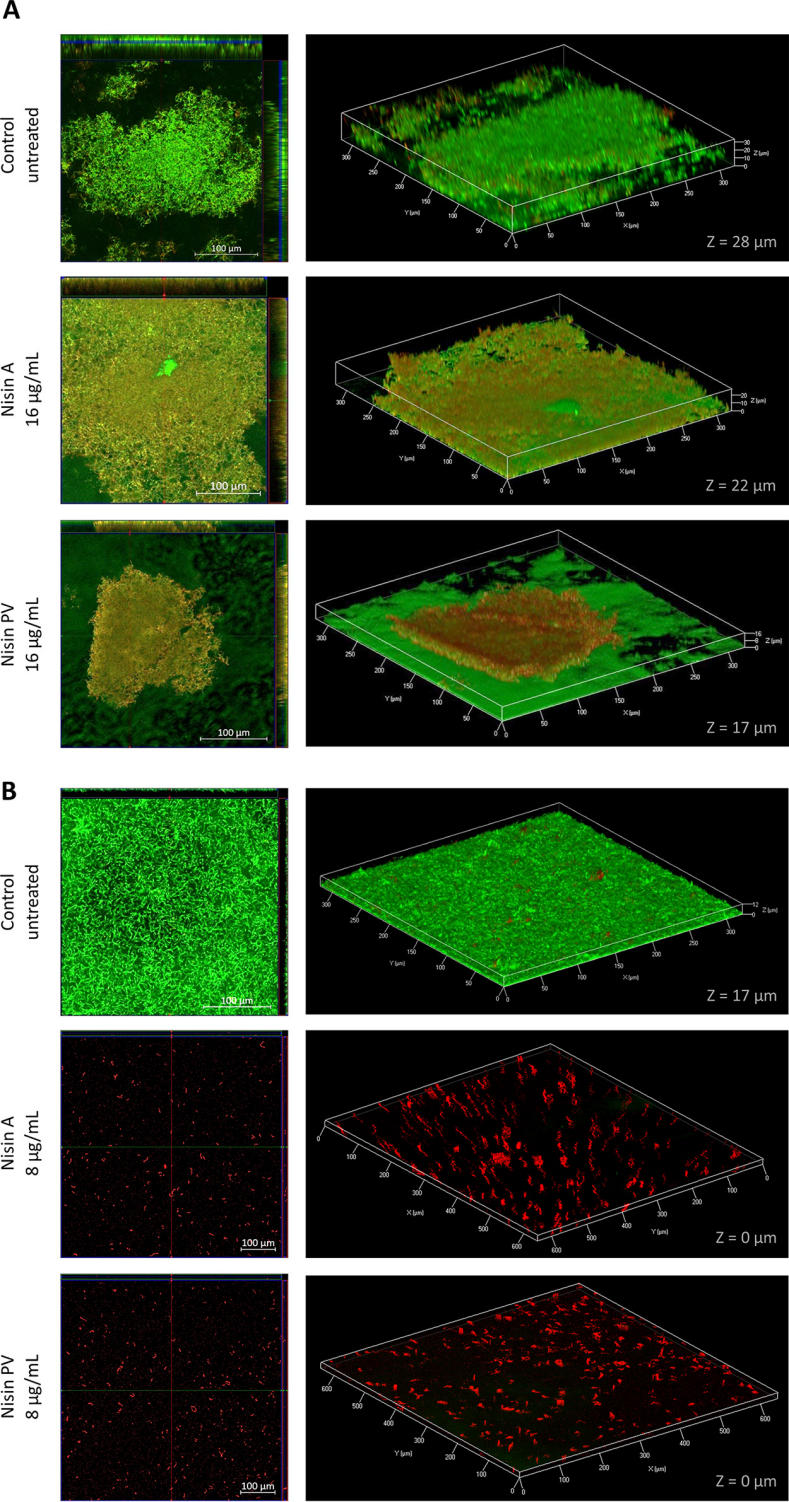
Confocal laser scanning microscopy (CLSM) biofilm images of *S. uberis* DPC 5344 (A) and *S. uberis* ATCC 700407 (B) after a 24-h treatment of a preformed biofilm with nisin A and PV (16 μg/ml for *S. uberis* DPC 5344 and 8 μg/ml *S. uberis* ATCC 700407, respectively). Cells were stained with SYTO 9 and propidium iodide (PI). The live cells are shown in green and the dead cells in red. The middle panel from the picture on the left side represents the *x*–*y* plane, and the adjacent top and side panels represent the *x*–*z* and *y*–*z* planes, respectively. On the right side, live/dead 3D CLSM images of biofilm eradication are shown. Z, thickness of the biofilm (μm).

Following treatments, the biofilms were visualized by confocal laser scanning microscopy (CLSM). The confocal microscopy was performed using LIVE/DEAD BacLight staining, which enables not only examination of the biofilm viability but also the architecture of the biofilm. The nisin concentration used for each strain was selected according to the highest concentration that presented a significant or large difference in biofilm eradication between nisin A and PV in the 2,3-bis-(2-methoxy-4-nitro-5-sulfophenyl)-2H-tetrazolium-5-carboxanilide (XTT) assay. Therefore, the ability of nisin A and PV to remove established biofilms of *S. uberis* DPC 5344 and ATCC 700407 was evaluated at concentrations of 16 μg/ml and 8 μg/ml, respectively. Photomicrographs following treatment of established *S. uberis* DPC 5344 biofilm with 16 μg/ml of nisin A and PV were taken by confocal microscopy and are shown in Fig. 4A. The control biofilm without any treatment reveals a biofilm with a thickness (Z) of 28 μm with a predominance of live cells in most of the structure. A few dead cells were found in the surrounding edges of the structure and where the biofilm thickness was decreased. The nisin A treatment displayed live and dead cells within the stack of the biofilm, but with a large percentage of dead cells, especially on the top. Following treatment with nisin A, a slight reduction of 4 μm in the biofilm thickness was found compared with the untreated control. Conversely, treatment with nisin PV revealed a reduced biofilm mass in the well and the presence of predominantly dead cells on the top of the structure. In addition, biofilm thickness was considerably reduced from 28 μm (untreated control) to 17 μm (nisin PV treatment).

In the case of *S. uberis* ATCC 700407-treated biofilms (Fig. 4B), although there was no significant difference between treatment with nisin A and PV, the concentration of 8 μg/ml was selected for evaluation by CLSM. As in the crystal violet (CV) assay and XTT assay, treatment with nisin PV revealed no difference in biofilm eradication activity compared to that with the use of nisin A. However, both nisin peptides were able to remove most of the biofilm from the well, with only a few red-stained cells found along the well.

### Architecture and viability of *S. uberis* biofilm after nisin treatment to prevent the biofilm formation evaluated by CLSM.

**FIG 5:**
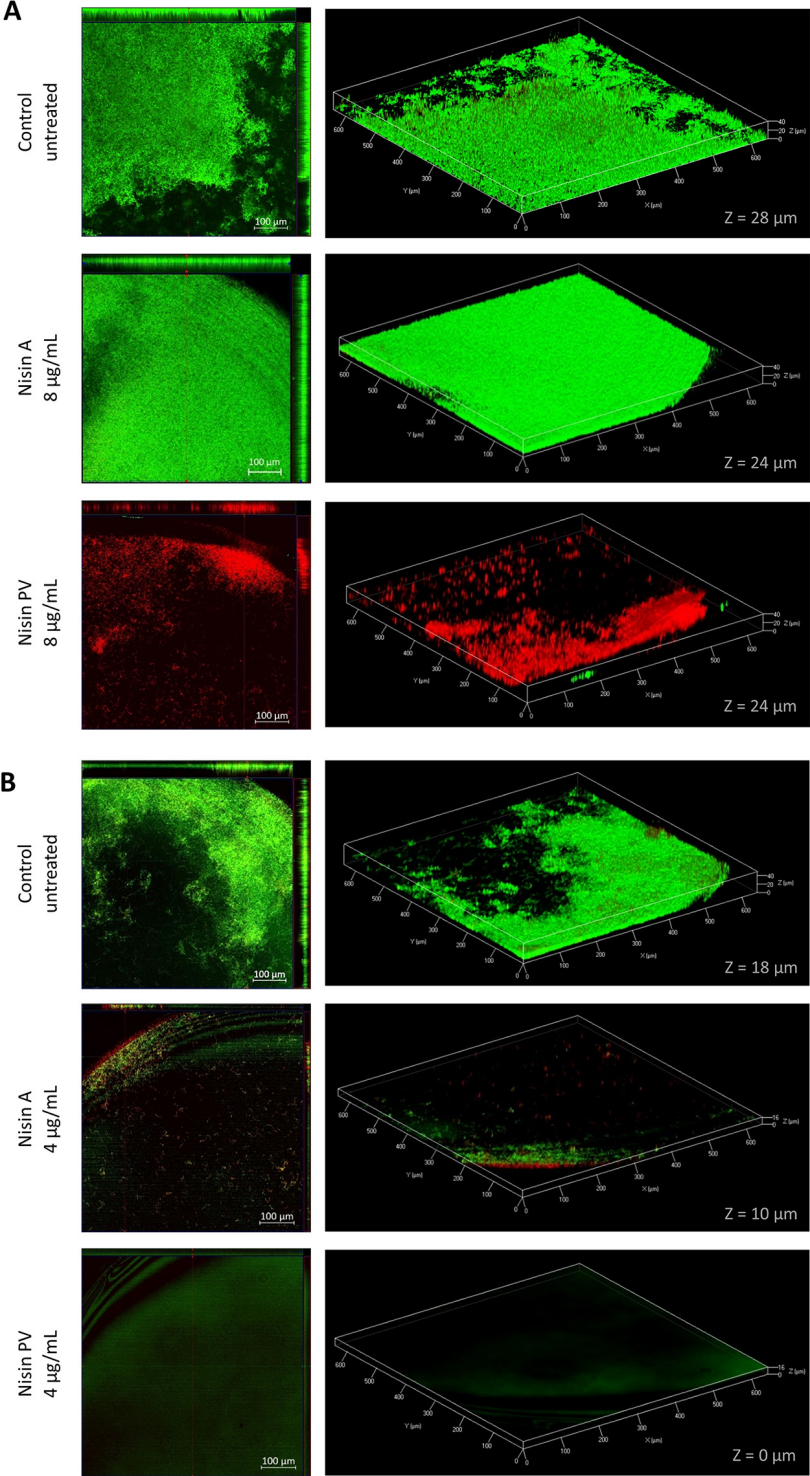
Confocal laser scanning microscopy (CLSM) biofilm images of *S. uberis* DPC 5344 (A) and *S. uberis* ATCC 700407 (B) following treatment for 24 h with nisin A and PV (8 μg/ml for *S. uberis* DPC 5344 and 4 μg/ml for *S. uberis* ATCC 700407, respectively). Cells were stained with SYTO 9 and PI. The live cells are shown in green and the dead cells in red. The middle panel from the picture on the left side represents the *x*–*y* plane, and the adjacent top and side panels represent the *x*–*z* and *y*–*z* planes, respectively. On the right side, live/dead 3D CLSM images of biofilm inhibition formation are shown. Z, thickness of the biofilm (μm).

An assessment of nisin A and PV for the prevention of biofilm formation by *S. uberis* DPC 5344 and ATCC 700407 was evaluated at concentrations of 8 μg/ml and 4 μg/ml, respectively. CLSM pictures taken of *S. uberis* DPC 5344 biofilms following treatments with and without nisin A and PV are shown in Fig. 5A. The biofilm thickness in the control was 28 μm, and only live cells were present. Treatments of *S. uberis* DPC 5344 with nisin A brought about a small reduction in biofilm thickness, estimated to be approximately 4 μm less than that of the nontreated control. Notably, although treatment with nisin PV also brought about only a slight reduction in biofilm thickness, comparable to that produced by nisin A, only dead cells were observed throughout the biofilm structure.

In contrast, *S. uberis* ATCC 700407 exposed to nisin revealed a significant reduction in biofilm formation to such an extent that, in the nisin PV treatment, the strain was incapable of adhering to the well. Therefore, nisin PV has the capacity to completely prevent biofilm formation at a concentration of 4 μg/ml (Fig. 5B).

## DISCUSSION

*S. uberis* is one of the most predominant pathogens involved in bovine mastitis and in several countries of the world is responsible for as many as one-third of all clinical bovine mastitis cases (20, 21). The most common treatment for mastitis caused by this pathogen is the use of β-lactam antibiotics, such as penicillin G or penethamate, and where more aggressive therapy is required, third- or fourth-generation cephalosporins (8, 22, 23). Unfortunately, many mastitis-associated strains are becoming resistant to these antibiotics with several reports revealing reduced sensitivity or resistance (24–28).

In recent years, the potential of lantibiotics as antimicrobial agents has attracted attention due their high *in vitro* potency, excellent *in vivo* activities, and ability to rapidly damage target cells. The lantibiotic nisin was the first bacteriocin to be approved by the World Health Organization (WHO), Food and Agriculture Organization (FAO), and Food and Drug Administration (FDA) for use as a food additive to control problematic microorganisms. Moreover, nisin has potential for use in a wide range of medical applications (29–31). However, recent reports have emerged of pathogenic bacteria that possess protein defense mechanisms against lantibiotics even though the organism itself does not produce such an antimicrobial (32). One of these systems relies on the expression of dedicated enzymes that inactivate the lantibiotic peptide through proteolytic cleavages, such as the nisin resistance protein (NSR). Therefore, new inhibition mechanisms are required to address these resistance systems that disrupt the effective use of lantibiotics as therapeutics. The gene-encoded nature of nisin A allows it to be manipulated to modify its biological and physical properties (33, 34). In this way, using bioengineering technology, new nisin derivatives can be created to increase their inhibitory activity or to evade NSR proteolytic systems expressed by some species of the *Streptococcus* genus, such as *S. uberis* (18). Indeed, in this study, the nisin derivative PV exhibited an enhanced inhibitory activity against *S. uberis* DPC 5344 and ATCC 700407, both NSR-producing strains, as evaluated by deferred antagonism assay. These results are consistent with previous findings by Field and collaborators (18), where these strains exhibited increased susceptibility against this nisin variant.

One of the most recognized virulence factors in *S. uberis* is its ability to form biofilms (35). Cells within a biofilm have increased tolerance to antibiotics, thus biofilms are a major cause of persistent and recurring infections (36, 37). In this work, both *S. uberis* mastitis-associated strains possessed the ability to form biofilms at 37°C after 24 h and in tryptic soy broth supplemented with yeast extract and 1% of glucose (TSB-YEg) medium. However, *S. uberis* ATCC 700407 formed a weaker biofilm compared to *S. uberis* DPC 5344. This can be due to several factors, such as the composition of the medium, growth conditions, as well as surface adhesion, among others. In accordance with these results, several studies have demonstrated how *in vitro* biofilm production displays wide variation among isolates (6, 38).

Previous studies have demonstrated the enhanced susceptibility of planktonic cells of NSR-producing strains to nisin PV compared with that of the wild-type nisin peptide, nisin A (18, 39). Indeed, analysis of a collection of Streptococcus agalactiae isolates (planktonic cells) by Hayes et al. (39) found that 64.8% (79 of 122) were more sensitive to nisin PV compared with nisin WT. Similarly, Field et al. (18) demonstrated an increase of up to 20-fold in the specific activity of nisin PV compared with that of nisin A against a range of NSR^+^ strains. The authors suggest that the greater inhibitory effect of nisin PV is due to the higher bond energy between atoms of the amino acids at positions 28 (alanine) and 29 (proline) of this derivative compared to that of nisin WT (alanine and serine at positions 28 and 29, respectively). As a result, increased rigidity and inflexibility in the region around the nisin cleavage site renders the action of NSR less effective, and consequently, PV displays higher bioactivity against microorganisms possessing this kind of resistance.

As outlined previously, nisin resistance in many streptococci is conferred by a proteogenous resistance system comprising NSR and an ABC transporter (NsrFP) belonging to the BceAB-type superfamily (11). Indeed, the genomes of *S. uberis* DPC 5344 and ATCC 700407 utilized in this study were found to encode both systems. Furthermore, it has been suggested that these two systems work cooperatively to provide full resistance (40). Moreover, studies with nisin mutants have revealed the importance of the N-terminal region of nisin for recognition by NsrFP from S. agalactiae (40, 41). Deferred antagonism assays carried out in our laboratory with a Lactococcus lactis strain expressing NsrFP revealed no difference in the bioactivity of nisin PV compared to that of nisin A (data not shown), suggesting that the potency of nisin PV is due to an exclusive evasion of the NSR system.

Numerous research studies have reported the antibacterial activity of nisin against other bovine mastitis pathogens, such as *Staphylococcus* spp., Staphylococcus aureus, Streptococcus agalactiae, Bacillus cereus, and Escherichia coli (42–45). Few studies have referred to the treatment of *S. uberis*, and even fewer have investigated its biofilm eradication (46, 47). Indeed, Montironi and collaborators (47) are the only group to date to have investigated the eradication and inhibition of some strains of *S. uberis* biofilms using essential oils and limonene derivates from the plant *Minthostachys verticillata*. However, to our knowledge, no studies have demonstrated the antibacterial activity of nisin against *S. uberis* biofilms. This study goes further and not only demonstrates the ability of nisin and nisin derivatives to inhibit and eradicate biofilms of *S. uberis* strains but also the inhibitory effect of nisin derivatives to target the biofilms of NSR-producing strains.

The ability of nisin PV to more effectively prevent and remove the biofilms of *S. uberis* strains encoding an NSR system than WT nisin was visibly demonstrated by the following three experiments performed in this study: CV assay, XTT assay, and CLSM.

In the biofilm eradication strategy, as expected, the concentration of nisin required to remove the preformed biofilm (or eliminate the presence of living cells in the biostructure) was higher than that required to inhibit its formation. With respect to *S. uberis* DPC 5344, concentrations of 16 μg/ml of both nisin A and PV were required to significantly reduce the presence of biofilm compared to the control. However, as observed in the XTT assay, treatment at this concentration of nisin PV brought about a significant reduction in metabolic activity of the cells in the biofilm compared to treatment with nisin A (Fig. 2B). These results are consistent with those obtained by confocal microscopy, where a significant reduction in the thickness of the biofilm was observed when treated with the nisin derivative compared to the control and the nisin WT treatment (Fig. 4A). Although both peptides exhibited the ability to reduce biofilm mass of *S. uberis* DPC 5344, treatment with nisin PV far exceeded that of nisin A, highlighting the potential of the nisin PV derivative against NSR^+^ *S. uberis* strains. Analysis of the effects of nisin A and nisin PV on the biofilm of *S. uberis* ATCC 700407 revealed complete removal of this structure at concentrations of 64 μg/ml as determined by crystal violet staining (Fig. 2C). Although a concentration of 32 μg/ml of either nisin peptide was adequate to achieve significant reductions in biofilm viability compared to that of the control as observed by the XTT assay, nisin PV outperformed nisin A at both 16 and 8 μg/ml (Fig. 2D). It is likely that the effective eradication of preestablished biofilm of this strain with both nisin A and nisin PV is due to the weaker nature of the biofilm formed by *S. uberis* ATCC 700407 (at least under the experimental conditions used for this work). The CLSM photography revealed the presence of a few dead cells of *S. uberis* ATCC 700407 adhering to the surface when treated with only 8 μg/ml of both nisin peptides (Fig. 4B). These results are in contrast to the results obtained by Corbin et al. (48), where eradication treatments using an antimicrobial solution of 0.005% nisin A on an oral biofilm community of *Streptococcus* and *Actinomyces* (Streptococcus oralis ATCC 10557, Streptococcus gordonii ATCC 10558, and Actinomyces naeslundii ATCC 19039) did not bring about any removal of the biofilm.

For the inhibition of biofilm formation by *S. uberis* DPC 5344, concentrations of 16 μg/ml of both nisin peptides completely inhibited the presence of metabolically active cells within the biofilm of this strain (Fig. 3B). These results are in accordance with other studies where nisin A was able to prevent the biofilm formation of other mastitis-associated strains, such as S. aureus (49), or saliva-derived multispecies biofilms, such as S. gordonii, S. oralis, and Streptococcus mutans (50). However, in our work, just 8 μg/ml of nisin PV was sufficient to obtain the equivalent inhibitory effect produced by using twice that of the parental nisin A. These results are in agreement with those published by Field et al. (18) and Hayes et al. (39), where lower concentrations of nisin PV were able to more effectively inhibit planktonic cell growth of NSR-producing strains. The treatment of *S. uberis* DPC 5344 with 8 μg/ml of nisin A revealed inhibition in the formation of the biofilm in comparison to that of the control, mainly by reducing the thickness of the biofilm from 28 μm to 24 μm as observed by CLSM (Fig. 5A). However, as observed by XTT and CLSM assays, the presence of abundant live cells was detected around the whole biofilm. In contrast, Angelopoulou and collaborators (49) reported that nisin A treatment of one mastitis-associated strain of S. aureus (APC3912CM) under investigation did not bring about any influence on the thickness of the biofilm but did on another strain of S. aureus (APC3814H). These results highlight the strain-dependence of the action of nisin A on different biofilms. Although the bacteria in this study were capable of adhering to the polystyrene surface (detected by CV), 8 μg/ml concentration of nisin PV was adequate to completely inhibit the presence of live cells in the biofilm (as detected by XTT). These results are consistent with images taken by confocal microscopy, where the presence of red-stained cells (dead cells) was in abundance. When the ATCC strain was assessed, both nisin A as well as PV were proficient in preventing the formation of biofilm by this strain. The treatments not only reduced the presence of live cells in the structures but also fully inhibited their formation. However, even at the lowest concentration of nisin PV tested (2 μg/ml) no metabolically active cells of *S. uberis* ATCC 700407 were detected during biofilm formation as observed by the XTT assay. Considering the confocal microscopy photographs of the samples treated at 4 μg/ml of both nisin peptides, treatment with nisin A resulted in the presence of a weak biofilm with a thickness reduction of nearly 44% compared to the untreated control (about 18 μm). However, treatment with the same concentration of nisin PV resulted in the complete inhibition of biofilm formation. These results demonstrate once again the higher inhibitory effectiveness of the nisin derivative PV over and above the native nisin, revealing that its activity is maintained even when applied in the treatment of biofilms of NSR-producer strains. Zhao and collaborators demonstrated that the treatment of S. mutans biofilm with nisin A (when incorporated in a dental adhesive product) caused a substantial inhibition in the growth of this microorganism as a biofilm (51). Indeed, they not only demonstrated the effectiveness of inhibiting S. mutans biofilm formation but also the successful effects on a multispecies biofilm.

Several studies have shown that the susceptibility of mastitis-associated strains to conventional treatment with penicillin G is considerably reduced under biofilm conditions (52–54). This highlights the requirement for higher concentrations of antibiotics in their inhibition and eradication. However, this is counterproductive in the current times where a conscious and reduced use of antibiotics is required to diminish the problems of emerging microorganisms resistant to antibiotics. Therefore, this work not only presents nisin A and PV as potential alternatives to the use of antibiotics but also demonstrates their effectiveness in the removal and prevention of biofilms of *S. uberis* strains with the ultimate aim of reducing the use of traditional antibiotics.

In conclusion, we show the effect of a novel nisin derivative, nisin PV, and wild-type nisin A on NSR^+^ *S. uberis* biofilms. Importantly, the biofilm inhibition and eradication efficacy of nisin PV exceeded that of nisin A in both tested strains, demonstrating that this nisin derivative remains more active even against the biofilms of NSR-producer strains.

## MATERIALS AND METHODS

### Microorganisms and culture conditions.

L. lactis NZ9800(pCI372-*nis*A) and L. lactis NZ9800(pCI372-*nis*A-PV) were used to produce nisin A and nisin PV, respectively. The cultures were grown in M17 broth supplemented with 0.5% glucose (GM17) and stored at −20°C in the same medium with 50% vol/vol glycerol. The NSR-producing strains *S. uberis* DPC 5344 (a mastitis-associated strain) and *S. uberis* ATCC 700407 (reference strain) were used to evaluate the competence of both nisin peptides to eradicate/inhibit their biofilms. Each *S. uberis* strain was cultivated in tryptic soy broth (TSB) (Merck, Germany) supplemented with yeast extract (YE) (Sigma-Aldrich) and 1% of glucose (TSB-YEg), incubated at 37°C for 16 to 18 h before use in each assay, and stored at −20°C in the same culture medium with 50% vol/vol glycerol (Table 1).

**TABLE 1:** Microorganisms used in this study

Strain	Relevant characteristic(s)	Reference or source
L. lactis NZ9800(pCI372-nisA)	Nisin A-producing strain	16
L. lactis NZ9800(pCI372-nisA-PV)	Nisin PV-producing strain	18
Streptococcus uberis DPC 5344	Mastitis-associated strain, biofilm producer, and NSR producer	DPC^*a*^
Streptococcus uberis ATCC 700407	Quality control reference strain, biofilm producer, and NSR producer	ATCC

a DPC, Teagasc Culture Collection, Moorepark Teagasc Food Research Centre, Fermoy, Co. Cork, Ireland.

### Deferred antagonism assay.

Deferred antagonism assays were performed to evaluate the inhibition ability of nisin A and nisin derivative PV against the *S. uberis* strains. Briefly, 10 ml of GM17 agar was added to a petri dish plate, allowed to set, and then inoculated with a 5-μl drop of an overnight culture of L. lactis NZ9800(pCI372-*nis*A) (nisin A producer) and L. lactis NZ9800(pCI372-*nis*A-PV) (nisin PV producer). The plate was incubated at 30°C overnight. After this time, the colonies were treated with UV light for 15 min to kill live cells. Once treated, 15 ml of TSB-YEg soft agar inoculated with 50 μl of an overnight culture of the relevant *S. uberis* strain was overlaid on the L. lactis colonies. The double-layer plates were incubated for 24 h at 37°C. The inhibition activity was observed by comparing the halo size around the L. lactis colony producers of nisin A and nisin PV.

### Nisin purification.

Tryptone yeast (TY) broth (2.4 liters) was prepared using tryptone (7.5 g), yeast extract (15 g), MnSO_4_·H_2_O (150 mg), and MgSO_4_ (375 mg). Prior to inoculation with the appropriate nisin producer, the broth was passed through a column with a length of 70 cm and an internal diameter of 5 cm (Bio-Rad Laboratories, CA, USA), and up to one-third of its height was filled with Amberlite XAD-16 beads (Sigma-Aldrich, Darmstadt, Germany). First, the beads were washed by passing water through the column (approximately 1 liter). After the washing step, 500 ml of broth was added to the column, allowed to pass through the beads, and discarded before proceeding with the rest of the culture medium. Subsequently, the rest of the broth was filtered and collected in bottles to be autoclaved at 121°C for 20 min.

Nisin A and the nisin PV derivative were purified according to previously described protocols (18). Briefly, 900 ml of the previously treated TY broth, supplemented with 50 ml of 200 g/liter glucose and 50 ml of 380 g/liter β-glycerophosphate, was inoculated with 1% of an overnight culture of the producing strain and incubated for 1 h at 30°C. After this time, 80 μl of a 0.1% Nisaplin solution was added to the culture to induce peptide production. The culture was incubated again for an additional hour and, subsequently, 800 μl of the Nisaplin solution was added and was kept at 30°C for 20 h. Following this, the culture was centrifuged for 20 min at 7,000 rpm. The supernatant was recovered and passed through 60 g of preequilibrated Amberlite XAD-16 beads. The beads were washed with 500 ml 30% ethanol, and the nisin from the beads was eluted using 500 ml 70% isopropanol (IPA) (Fisher Scientific, MA, USA) and 0.1% trifluoroacetic acid (TFA) (Sigma-Aldrich, Darmstadt, Germany). Concomitantly, the cell pellets were resuspended in 300 ml of 70% IPA and 0.1% TFA and stirred at room temperature for 3 h followed by centrifugation. This cell supernatant was combined with that referred to above and was concentrated through rotary evaporation (Büchi, Switzerland) to approximately 250 ml. Following pH adjustment to 4.0, further concentration was achieved using a Phenomenex SPE C_18_ column to a final volume of 60 ml. Eleven milliliters of this sample was concentrated again through rotary evaporation to 2 ml and applied to high-pressure liquid chromatography (HPLC) using a Phenomenex C_12_ reverse-phase (RP) HPLC column (Jupiter 4 μm proteo 90 Å, 250 by 10.0 mm, 4 μm). To facilitate this, a gradient of 30 to 50% acetonitrile (Fisher Scientific, MA, USA) containing 0.1% TFA was settled. The relevant fractions were collected and pooled, subjected to rotary evaporation to remove acetonitrile and subsequently freeze-dried (Labconco, MO, USA). The purified peptides were subjected to matrix-assisted laser desorption ionization–time of flight (MALDI-TOF) mass spectrometric analysis to confirm their purity before use.

### Mass spectrometry.

Mass spectrometry of the purified peptide resuspension was performed with an Axima TOF2 MALDI TOF mass spectrometer (Shimadzu Biotech, Manchester, UK). Matrix solution (alpha-cyano-4-hydroxy cinnamic acid [CHCA], 10 mg ml^−1^ in 50% acetonitrile 0.1% [vol/vol] trifluoroacetic acid) was placed on the target for 60 s and then removed. The remaining solution was then allowed to air dry, and the sample solution was placed onto the precoated sample spot. Following addition of 0.5 μl matrix solution and air-drying, the sample was subsequently analyzed in positive-ion reflectron mode.

### Biofilm formation.

Biofilm formation by *S. uberis* strains ATCC 700407 and DPC 5344 was set up in microtiter plates based on a previous study (55) with modifications. Briefly, overnight cultures from TSB-YEg were used to prepare a 1:100 dilution as inoculum. Two hundred microliters of this dilution was transferred to wells of a sterile 96-well microtiter plate (Sarstedt, Leicester, UK) to obtain a starting inoculum of 10^5^ CFU ml^−1^; 200 μl of TSB-YEg was added to a set of wells as negative controls. The plates were incubated without shaking at 37°C for 24 h to allow biofilm formation.

### Biofilm eradication by nisin A and nisin PV.

The effect of increasing concentrations of nisin peptides on biofilms of *S. uberis* DPC 5344 and ATCC 700407 was evaluated as representative NSR and mastitis-associated strains. Established biofilms were washed once with phosphate-buffered saline (PBS), and 200-μl solutions of nisin peptides were added separately to the microtiter plate wells at 2, 4, 8, 16, 32, 64, and 128 μg/ml of nisin A or PV corresponding to 1/8×, 1/4×, 1/2×, 1×, 2×, 4×, and 8×, respectively, of the MIC value previously determined for the nisin A peptide on planktonic culture (18). Wells with *S. uberis* biofilms or medium alone were used as positive and negative controls, respectively. The plate was incubated at 37°C for 24 h, and following incubation, the culture in the wells was removed and gently washed with PBS. The nisin-treated biofilm was evaluated for quantity, viability, and architecture by CV assay, XTT assay, and confocal laser scanning microscopy (CLSM), respectively. All of the experiments were performed by triplicate in three biological replicates.

### Biofilm inhibition by nisin A and nisin PV.

For the evaluation of biofilm inhibition, the biofilm formation assays were performed as described previously with minor modifications. TSB-YEg supplemented with nisin A or PV at 2, 4, 8, and 16 μg/ml separately was added to wells containing the *S. uberis* strains at 10^5^ CFU/ml and incubated at 37°C for 24 h. Cultures of *S. uberis* alone were used as controls. After this time, the biofilm was washed with PBS, and the CV assay, XTT assay, and CLSM were then performed to estimate bacterial biomass, cell viability, and architecture, respectively. All of the experiments were performed in triplicate in three biological replicates.

### Crystal violet assay.

To quantify biofilm formation, the culture was carefully removed and the wells washed with PBS. The remaining attached bacteria were fixed with 200 μl methanol for 15 min. Afterward, the methanol was removed and let dry for 5 min. The biofilm was stained for 15 min with 200 μl 0.05% (wt/vol) crystal violet. Excess stain was rinsed twice with 200 μl PBS per well. After the wells were air-dried, the dye bound to the adherent cells was dissolved with 200 μl 33% (vol/vol) acetic acid and placed on a shaking plate for 30 min at 100 rpm. The optical density (OD) of 100 μl of each well was measured at 595 nm using a microplate reader (Spectramax M3; Molecular Devices, Sunnyvale, CA, USA). Wells filled with growth medium were included as negative controls. Data obtained in triplicate were calculated and expressed as the mean ± standard deviations. The biofilm ability of the strains was evaluated following the Stepanović criteria (19). According to this, the optical density cut-off value (ODc) was defined as 3 standard deviations above the mean optical density (OD) of the negative control. The following classification was used for the determination of *in vitro* biofilm formation: nonbiofilm producer when OD < ODc, weak biofilm producer when ODc < OD < 2× ODc, moderate biofilm producer when 2× ODc < OD < 4× ODc, and strong biofilm producer when 4× ODc < OD.

### XTT viability assay.

Cell viability of treated biofilms was evaluated by the XTT assay. XTT is a tetrazolium derivative that produces an orange-colored formazan product when cleaved by mitochondrial dehydrogenase in viable cells (56). The XTT solution was prepared by dissolving 0.5 mg XTT in 1 ml of PBS and then supplementing it with 2.5 μl of a 10 mM menadione stock solution (dissolved in acetone). A 200-μl volume of XTT-menadione solution was added to each well. Plates were incubated in the dark for 3 h at 37°C. One-hundred-microliter volumes of the supernatant were transferred to the wells of a new 96-well flat-bottom plate, and the absorbance at 490 nm was measured with a microplate reader (Spectramax M3; Molecular Devices, Sunnyvale, CA, USA). Data obtained in triplicate were calculated and expressed as the mean ± standard deviations.

### Confocal laser scanning microscopy.

*S. uberis* biofilms treated with nisin A and PV were visualized by CLSM. In this case, the biofilms were preformed, and treatment was as described previously performed on μ-Slide 8-well uncoated microtiter plates (Ibidi, Germany) suitable for confocal microscopy applications. After the peptide treatment, adherent bacteria were rinsed once with PBS and stained using a LIVE/DEAD BacLight bacterial viability kit (L7012; Invitrogen) according to the manufacturer’s instructions. Two hundred microliters of the 0.3% solution containing SYTO 9 and propidium iodide (PI) mixed in a ratio of 1:1 in PBS was added to the biofilms. The μ-Slide 8-well microtiter plate was incubated at room temperature for 15 min in the dark. After incubation, the residual stain was removed from the wells and 100 μl of PBS was added. The biofilms were analyzed using a Zeiss LSM 5 confocal microscope with EC Plan-Neofluar 20×/0.5 M27 lens. SYTO 9 fluorescence, corresponding to live bacteria, was acquired in the green channel (475 to 525 nm), and propidium iodide fluorescence, which does not penetrate viable bacterial cells, was acquired in the red channel (566 to 719 nm). Images were acquired using the Zen 3.0 software and were used to evaluate the thicknesses of the three-dimensional biofilm images acquired by CLSM.

### Statistical analysis.

Data were statistically analyzed using IBM SPSS Statistics Software v.26.0 (IBM Corp., Armonk, NY, USA) and InfoStat v.18 (Centro de Transferencia InfoStat, FCA, Universidad Nacional de Córdoba, Argentina). To evaluate significant differences among samples, *t* test analysis was used. A *P* value of <0.05 was considered statistically significant.
